# The Effect of Month of Harvesting and Temperature–Humidity Index on the Number and Quality of Oocytes and In Vitro Embryo Production in Holstein Cows and Heifers

**DOI:** 10.3390/biology12091174

**Published:** 2023-08-26

**Authors:** Juan L. Morales-Cruz, Guadalupe Calderon-Leyva, Oscar Angel-García, Juan M. Guillen-Muñoz, Zurisaday Santos-Jimenez, Miguel Mellado, Luiz Gustavo Pessoa, Hugo Z. Guerrero-Gallego

**Affiliations:** 1 Departamento de Ciencias Veterinarias, Universidad Autónoma Agraria Antonio Narro-Unidad Laguna, Torreón 25315, Mexico; moralesnarro@hotmail.com (J.L.M.-C.); gcalderon06@hotmail.com (G.C.-L.); mvz.oscar_2207@hotmail.com (O.A.-G.); jmanuel.guillenm@uaaan.edu.mx (J.M.G.-M.); mvz_zusan@hotmail.com (Z.S.-J.); 2 Departamento de Nutrición Animal, Universidad Autónoma Agraria Antonio Narro, Saltillo 25315, Mexico; mmellbosq@yahoo.com; 3 Independent Researcher, Torreón 25315, Mexico; luizgustavo.rocha@genusplc.com

**Keywords:** heat stress, temperature–humidity index, dairy cows

## Abstract

**Simple Summary:**

There are several factors that can affect embryo production; among the most important causes is heat stress (HS), which is related to a decrease in the quality and number of embryos produced. HS occurs when animals are exposed to temperatures that exceed their thermal comfort threshold or when body temperature rises or falls from the thermoneutral zone (TNZ) of the animal. It has been observed that a high ambient temperature can cause reversible or irreversible cell damage in different structures and organelles of the embryo. However, there is no information on which month of the year can most negatively affect embryo production. In this sense, the results obtained in our research reaffirm that high-yielding dairy cows in a hot-arid environment at 25 °N are susceptible to heat load, which is reflected in the lower number and quality of oocytes harvested by ovum pick-up and the in vitro resultant embryos.

**Abstract:**

The aim of this study was to evaluate the effect of the month of oocyte harvesting and the temperature–humidity index on oocyte number and quality harvested from Holstein cows and heifers, oocyte developmental competence, and total embryos produced in an area of intense ambient temperature for most of the year. A total of 5064 multiparous lactating cows and 2988 nulliparous heifers were used as oocyte donors and distributed across the months of the year. Overall, total oocytes per collection did not differ (*p* > 0.05) between cows (16.6 ± 2.7) and heifers (15.1 ± 1.8), but oocyte developmental competence was lower (*p* < 0.05) in cows (21.3 ± 5.4) than heifers (25.5 ± 4.0). For cows, the total number of oocytes harvested was two-fold higher (*p* < 0.05) in November than in May. For heifers, the total number of oocytes harvested was highest in April (17.19 ± 0.53) and lowest in May (10.94 ± 0.32; *p* < 0.05). For cows, total embryos were highest in November (2.58 ± 0.42) and lowest in August (1.28 ± 0.10; *p* < 0.05). Thus, taken together, these results indicate that severe heat stress impaired the number and quality of oocytes harvested from donor Holstein multiparous cows and heifers, oocyte developmental competence, and total embryos produced in this area of intense ambient temperature for most of the year.

## 1. Introduction

Heat stress (HS) can be defined as the inability of an animal to dissipate sufficient body heat and maintain homeothermy or thermal equilibrium, caused mainly by high air temperatures, high humidity, thermal radiation, poor air movement, and metabolic heat [1]. HS levels in cattle can be estimated by using the temperature–humidity index (THI), which was adapted to describe the ambient temperature and relative humidity that causes HS [2]. Over a number of years, several studies have revealed the importance of environmental conditions on the reproduction performance of dairy cows subjected to HS [1,3,4,5], which constitutes a significant challenge for the global milk production industry and the welfare of cows [6,7]. During the warm summer months, milk production decreases by 10 to 35%, which is a problem in the dairy industry [8]. Under these circumstances, dry matter intake is reduced, water consumption is increased, and nutrient absorption is reduced [9].

Currently, HS is of great concern due to its detrimental impact on reproductive efficiency, especially in high-milk-producing cows [10]. In relation to this, a group of researchers has shown that this deleterious effect is enhanced in high-producing cows compared to lower-producing cows or heifers [7,11]. Thermal stress occurs when animals are exposed to temperatures that exceed their thermal comfort threshold or when the body temperature rises or falls from the thermoneutral zone (TNZ) of the animal. In Holstein cows, the TNZ ranges from 5 °C to 25 °C [2]. The response to HS brings about physiological, metabolic, cellular, and molecular changes [4,12].

Holstein cows subjected to increased HS present reproductive problems reflected in their reproductive capacity and physiological and cellular functions in various tissues [6]. It has been shown that a high ambient temperature compromises follicular growth, hormonal secretion, steroidogenesis, as well as uterine and endometrial blood flow, causing a low potential in the development and quality of oocytes, which later compromises the embryo development [12,13]. Under in vivo conditions, embryonic development is affected by HS during days 1 to 7 after estrus [10]. It has been observed that a high ambient temperature can cause reversible or irreversible cell damage in different structures and organelles of the embryo [14,15]. Such effects can trigger an adaptive response and/or cell death (apoptosis) [12].

In some areas of Mexico, high ambient temperatures are typical during spring, summer, and autumn, which drastically reduces the reproductive performance of dairy cows [16]. It has been documented that the percentage of conception decreases from 40% obtained in the temperate or cold months of the year to 15% during the summer months [2,7]. This is possibly due to the detrimental effect of HS that alters the quantity and quality of oocytes and their ability to develop into embryos [17,18]. In this sense, Hansed [19] mentions that HS can affect the reproductive axis, even weeks before ovulation, by compromising follicular function and ovogenesis, which in turn compromises oocyte competence and therefore fertilization, resulting in embryonic development problems up to the blastocyst stage. Research related to the effect of HS on the conception rate of embryos developed by in vivo techniques in dairy cattle [20] and beef cattle [21] has been carried out. Unfortunately, these investigations, although they have contributed significantly to the understanding of the mechanisms related to embryonic development, present certain limitations because these evaluations have been carried out with embryos produced in vivo, but in relation to embryos developed and produced in vitro, the information is scarce. Payton et al. [22] evaluated the effect of HS on germinal vesicle development by culturing and maturing oocytes in vitro by inducing a temperature increase in laboratory conditions up to 41 °C, and determined that this increase had a deleterious effect on embryo quality. Currently, there is information regarding the effect of HS and the THI related to the time of year, reproduction, and embryo production [23,24,25]. However, there is a paucity of research on oocyte yield using the ovum pick-up (OPU) technique and in vitro embryo production. With this in mind, research conducted in our laboratory evaluated the effect of HS and the THI on in vitro embryo production (IVEP) at different times of the year and found that some seasons had a deleterious effect on oocyte competence [26].

Therefore, this study aimed to evaluate the effect of the month of oocyte harvesting and the intensity of HS (temperature–humidity index) at the time of collection of transvaginal oocytes by the OPU technique on the oocyte traits and their developmental competence for IVEP in Holstein cows and heifers subjected to HS for most of the year.

## 2. Materials and Methods

### 2.1. General

The handling of the animals used in this study was carried out following the guidelines for the ethical use of animals for research [27], national guidelines [28], and a review by the collegiate body of investigation at the Agrarian Autonomous Antonio Narro University, with reference number UAAAN-UL/1330-8241-2868. The techniques used in this research were approved by the Bioethics Committee and by the Internal Committee for the Care and Use of Laboratory Animals (CICUAL) of the Faculty of Medicine of the Autonomous of Coahuila University (No. CONBIOETI-CA07CEI00320131015).

### 2.2. Location of the Study Area and Climatic Conditions

The study was carried out on 5 commercial dairy farms in northern Mexico within a radius of 11 km of each other (25°31′ N and 103°13′ W), at an altitude of 1100 m above sea level. The region has a semi-arid climate with an average annual rainfall of 230 mm; the average maximum temperature is 41.4 °C during May and June, and a minimum of −2 °C during December and January. Relative humidity ranges between 20 and 55% throughout the year [29].

### 2.3. Meteorological Data and THI Calculation

Meteorological data were obtained from a climatological station located within a 5 km radius of the dairy farms. Thus, these data truthfully reflect weather conditions on the dairy farms. Climatic information consisted of daily maximum temperatures in °C and relative humidity. In addition, the air temperature was recorded with a mercury thermometer shielded from radiation and moisture but freely exposed to the air. This information was used to calculate the daily temperature–humidity index (THI) using the following equation [30]:

THI = 0.8 × temperature + RH/100 × (temperature − 14.4) + 46.4.

RH refers to relative humidity. According to Mader et al. [30], Armstrong [31], and Lopez et al. [32], THI < 71 is considered as a thermal comfort zone, 72 to 78 is considered as mild HS, 79 to 88 is severe HS, and >89 is extreme HS (Table 1).

### 2.4. Animals and Handling Conditions

A total of 8052 Holstein cows were used, with a body condition score between 3.25 and 3.75 on a scale of 1–5 [33]; 5064 were multiparous cows, and 2988 were heifers. All donor females were gynecologically reviewed and only cows that did not present reproductive disorders according to gynecological examination were selected. The oocyte donor females were housed in open pens of 60 × 60 m corresponding to the intensive system. The corrals contained shades areas in the middle of the corral with a size of 9 × 30 m and orientation N-S. In addition, each corral had a feeder and a clean water supply freely accessible. The pens were also equipped with a cooling system of four 64 cm diameter fans installed at a height of 3.5 m from the ground, with four 25 mm diameter nozzles that sprayed 7 L of water/hour each or a total of 28 L/hour. The fans were of a power of 1.1 HP at 1000 rpm, with an air velocity of 13.7 kph at 6 m distance [34].

Cows were selected based on a 305-d milk production greater than 10.000 kg in their previous lactation. Cows underwent oocyte collection between 60 and 90 days in milk (DIM). The feeding followed the NRC [35] requirements, corresponding to the physiological state and milk production of the cows. Consisting mainly of a commercial concentrate (700 g/kg) and alfalfa hay (300 g/kg), these diets were served twice daily (07:00 and 18:00).

### 2.5. Design of the Experiment

**Figure 1:**
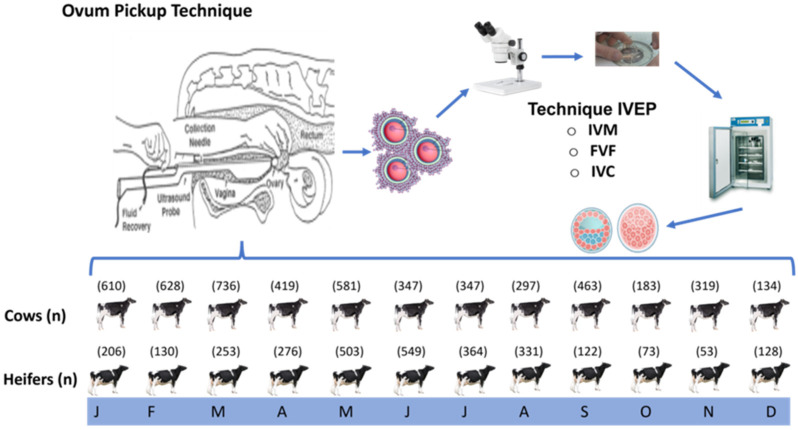
Scheme of the oocyte collection throughout the year using the ovum pick up (OPU) technique and the stages of the in vitro embryo production (IVEP) technique to which the oocytes from cows and heifers were subjected. IVM = in vitro maturation, IVF = in vitro fertilization, IVC = in vitro culture. Numbers in brackets are number of animals subjected to OPU per month. The months designated for each season of the year were the following: spring (March, April, May), summer (June, July, August), autumn (September, October, November), and winter (December, January, February).

The animals selected as oocyte donors were randomly distributed according to the days in milk during different months of the year in the case of multiparous cows, while for the group of heifers, animals were distributed according to the disposition of these during each month of the OPU (Figure 1).

The OPU was performed in vivo by a single qualified technician according to the technique described by Solís et al. [36], using ultrasound equipment (CHISON, Digital Ultrasound System, Model: 8300 VET. 5.0 MHz sector, Wuxi, China), connected to a vaginal transducer and coupled to a follicular aspiration guide with a cannula (20 G × 2″), attached to a suction circuit equipped with a 50 mL conical tube for the collection of liquid with a vacuum pump (Pionner Pro Pump, Pioneer Pro Pump Single −115 v, Single Foot Pedal, PS 653, Richmond, BC, Canada).

Subsequently, the cumulus–oocyte complexes (COC) collected in each OPU session were counted, morphologically evaluated, and classified based on their quality according to De Loos et al.’s [37] criteria. Oocytes of grades I, II, and III were considered viable, and grade IV was deemed non-viable. After evaluation, the COCs were subjected to the IVEP process according to the technique described by Paula-Lopes and Hansen [38]. For this process, 20 oocytes were placed in 50 µL microdroplets of maturation medium (TCM199 with Earle’s salts supplemented with 10% fetal bovine serum, 100 IU of penicillin mL^−1^, 0.01 mg of streptomycin mL^−1^, two µg of estradiol mL^−1^, 20 µg of FSH mL^−1^, and 0.2 mmol of sodium pyruvate mL^−1^) for 24 h at 37 °C with 5% CO_2_ with humidified air. After in vitro maturation, COCs were washed in Hepes-TALP medium and transferred to 4-drop capacity plates, in groups of 30 oocytes per drop, with fertilization medium containing 600 µL of IVF-TALP and 25 µL of PHE (0.5 mM penicillin, 0.25 mM hypotaurine, and 25 µM epinephrine in 0.9% NaCl [*w*/*v*]) and were fertilized with 1 × 106 Percoll-purified sperm cells under the same conditions as in vitro maturation. After 24 h, zygotes were separated from the surrounding cells by shaking them in Hepes-TALP medium for 5 min in microcentrifuge tubes and washed two or three times with Hepes-TALP. Then, the zygotes were placed in groups of 25 to 30 embryos in 50 µL drops of optimized modified potassium simplex medium, covered with mineral oil at 38 °C and 5% CO_2_ (*v*/*v*) in humidified air. The embryos obtained were morphologically evaluated according to what was described by Bó and Mappletoft [39].

The percentage of oocyte competence was defined as the ability of the oocyte to mature, undergo fertilization, and develop into embryo in vitro, 7 days after embryo culture [40].

### 2.6. Statistical Analysis

Data were tested for normal distribution using the Shapiro–Wilk test and homogeneity of variances was analyzed through a Bartlett test. Subsequently, the data were evaluated by ANOVA in a completely randomized design (PROC GLM). When there were statistically significant differences in the variables, comparison among months was carried out with the least significant difference after Bonferoni corrections. Associations between THI and total oocytes collected, viable oocytes, oocyte developmental competence, and total embryos were evaluated using non-linear models (CurveExpert Professional 2.5.6 4 software; Hyams Development, Madison, AL, USA). All results in the main text and figures are expressed as mean ± SD and statistical significance was accepted from *p* < 0.05.

## 3. Results

### 3.1. Total Oocytes

**Figure 2:**
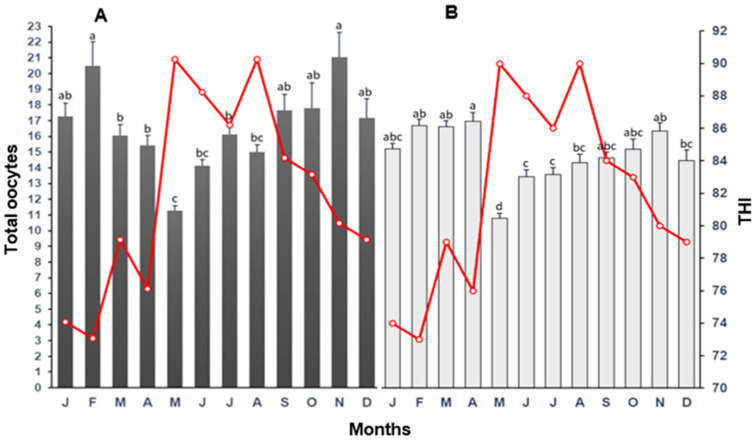
The mean (±SD) of total oocytes harvested following oocyte recovery procedure (OPU) from high-yielding multiparous cows (**A**) and heifers (**B**) in relation to THI (red line) on collection month. ^a–d^ = different letters indicate statistical difference (*p* < 0.05).

**Figure 3:**
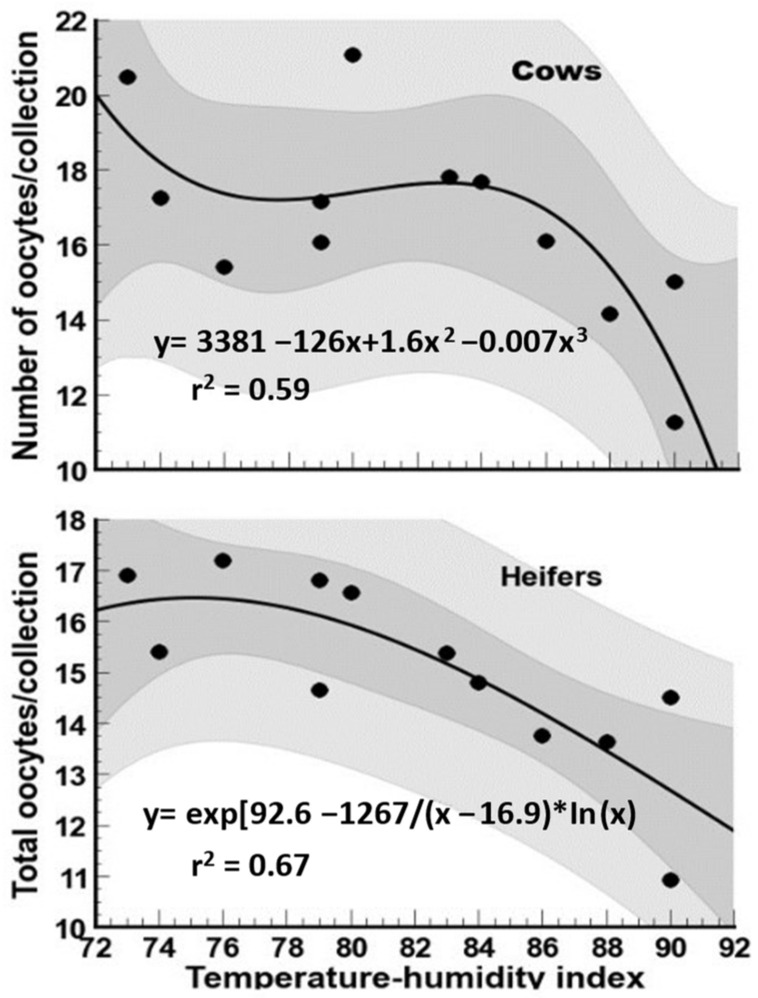
Association between temperature–humidity index (THI) and total oocytes harvested following oocyte recovery procedure (OPU) from high-yielding multiparous cows and heifers in a hot environment.

The influence of the THI in the month of oocyte collection in cows and heifers on the total number of oocytes harvested is presented in Figure 2. Total oocyte collection markedly decreased (*p* < 0.05) in May for both cows and heifers. The highest number of oocytes collected occurred in February and November for cows (*p* < 0.05) and February, March, April, and November for heifers (*p* < 0.05). For both cows and heifers, total oocytes/collection steadily increased from May to November. Total oocytes collected in November were two-fold higher than those collected in May. For heifers, there was a fairly strong association (*p* < 0.01) between total number of oocytes recovered and the THI (Figure 3), with a steady decline in the number of oocytes from THI = 76 onwards. In the case of cows, oocyte recovery plummeted at THI = 86.

### 3.2. Viable Oocytes

**Figure 4:**
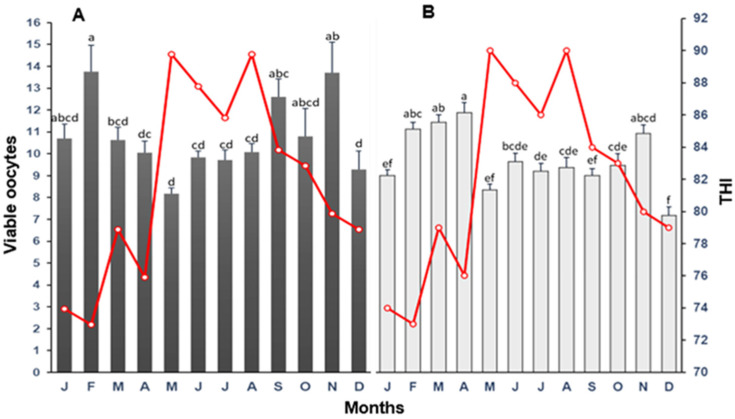
The mean (±SD) of total viable oocytes harvested following oocyte recovery procedure (OPU) from high-yielding multiparous cows (**A**) and heifers (**B**) in relation to THI (red line) on collection month. ^a–f^ = different letters indicate statistical difference (*p* < 0.05).

**Figure 5:**
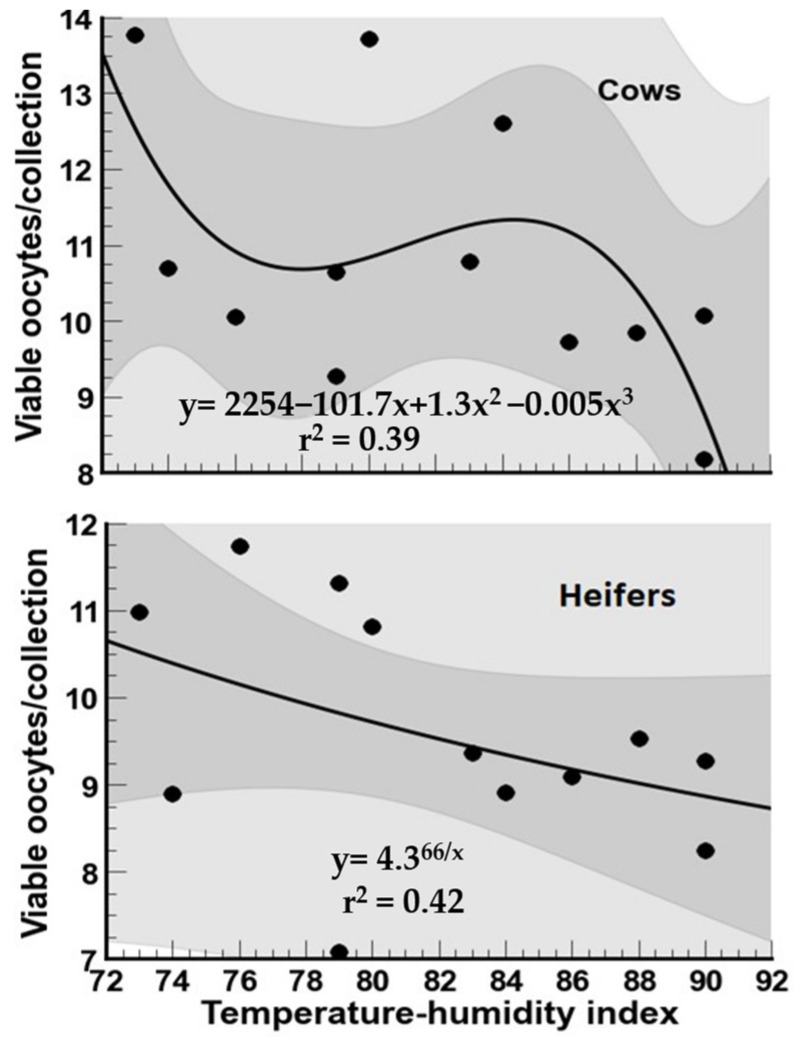
Association between temperature–humidity index (THI) and total viable oocytes harvested following oocyte recovery procedure (OPU) from high-yielding multiparous cows and heifers in a hot environment.

The mean population (±SD) of viable oocytes recovered from cows and heifers by OPU in relation to the THI by month of collection is presented in Figure 4. For cows, the general tendency (*p* < 0.05) was for lower numbers of viable oocytes to be obtained during the warmer (summer) months and, after that, a higher follicular population during the cooler (winter and autumn) months. For heifers, the months with the higher number of (*p* < 0.05) viable oocytes were February to April and November, with the lowest numbers in summer and autumn months. For cows, the association between the THI and viable oocytes was weak, without a clear tendency of HS on this variable (Figure 5). However, there was a tendency for lower viable oocytes for heifers as the THI increased (r^2^ = 0.42).

### 3.3. Oocyte Developmental Competence

**Figure 6:**
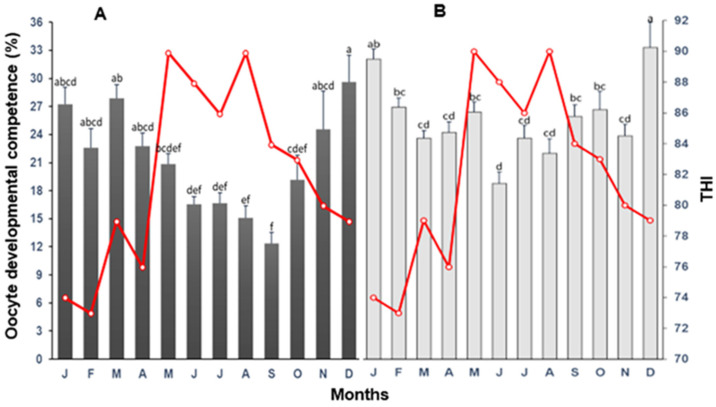
The mean (±SD) of oocyte developmental competence following oocyte recovery procedure (OPU) from high-yielding multiparous cows (**A**) and heifers (**B**) in relation to THI (red line) on collection month. ^a–f^ = different letters indicate statistical difference (*p* < 0.05).

**Figure 7:**
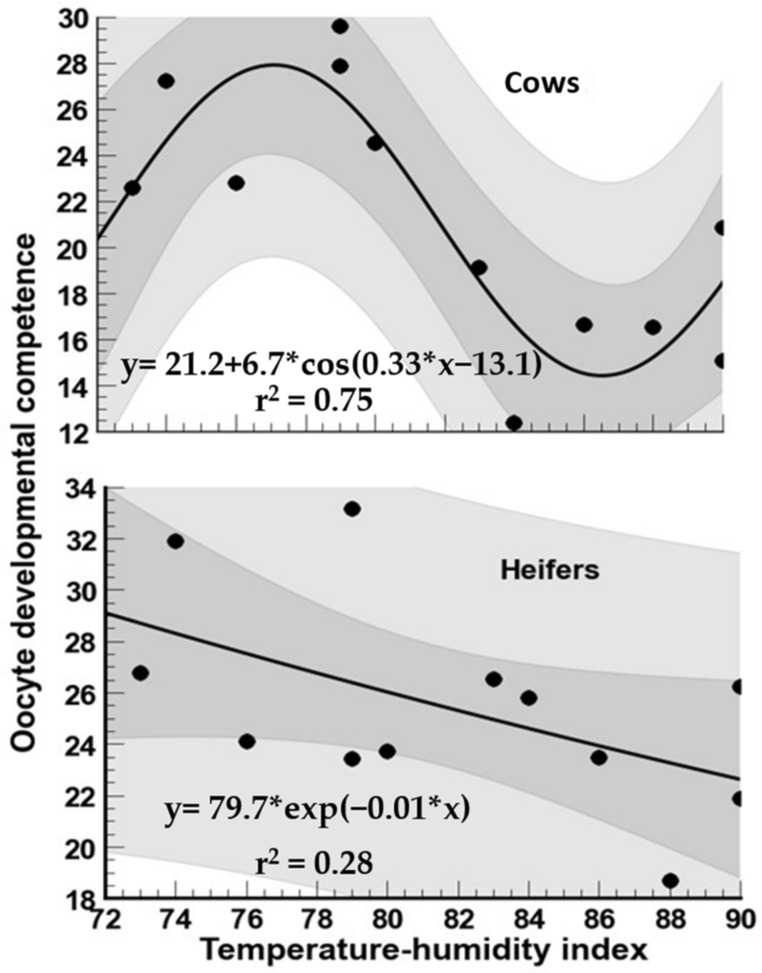
Association between temperature–humidity index (THI) and oocyte developmental competence following oocyte recovery procedure (OPU) from high-yielding multiparous cows and heifers in a hot environment.

For cows, a tendency for a decline (*p* < 0.05) in oocyte developmental competence was observed from January to September. Oocyte developmental competence was two-fold higher (*p* < 0.05) in December than in September. The highest values (*p* < 0.05) of competence in oocyte development were reached in heifers in January and December (THI between 72 and 79), and the lowest in the summer months (THI between 86 and 90; Figure 6). For cows, oocyte developmental competence declined markedly with THI > 80, whereas in heifers, a steady decline of this variable occurred from moderate (THI = 76) to severe (THI = 90) HS (Figure 7).

### 3.4. Total Embryos

**Figure 8:**
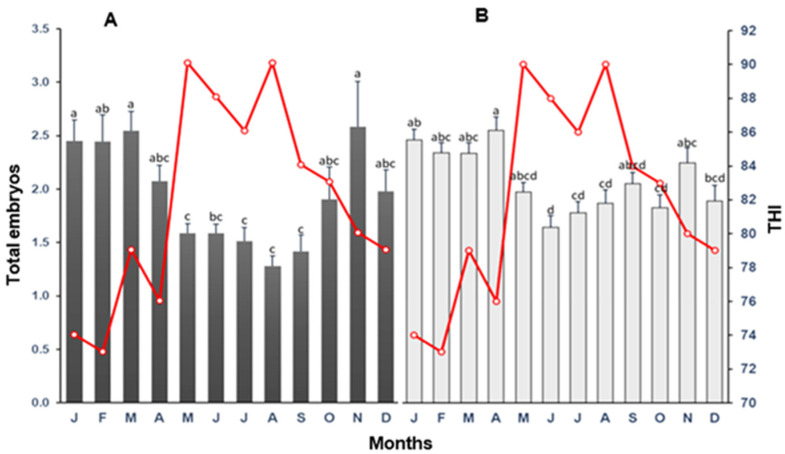
The mean (±SD) of total embryos derived from oocytes harvested following oocyte recovery procedure (OPU) from high-yielding multiparous cows (**A**) and heifers (**B**) in relation to THI (red line) on collection month. ^a–d^ = different letters indicate statistical difference (*p* < 0.05).

**Figure 9:**
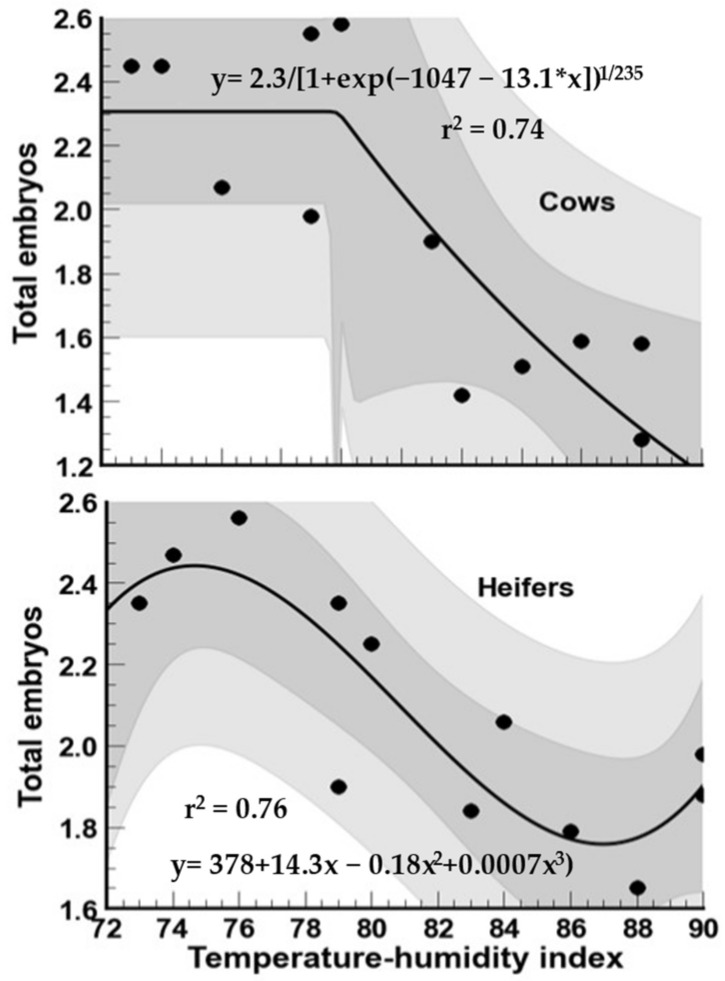
Association between temperature–humidity index (THI) and total embryos derived from oocytes harvested following oocyte recovery procedure (OPU) from high-yielding multiparous cows and heifers in a hot environment.

The mean (±SD) total embryo produced per collection in heat-stressed multiparous cows and heifers is presented in Figure 8. For cows, there was a significant difference (*p* < 0.05) in the number of embryos produced per OPU session for the other months of the year. The highest mean number of embryos per OPU session was recorded in the winter months and November, whereas the lowest values were registered in the summer months. The same tendency was observed for heifers, although the reduction in embryo production in summer was not as pronounced as in multiparous cows. In cows, embryo production markedly declined when the THI reached 80 units, whereas, in heifers, there was a decline in embryo production from THI = 78 (Figure 9). However, surprisingly, the lowest embryo production did not coincide with the highest THI (90 units).

## 4. Discussion

### 4.1. Total Oocytes

In the present study, the total number of oocytes obtained in cows and heifers was lower in the hottest months. May was the lowest average number of total oocytes produced in both groups of animals. This is in line with Rust et al. [41], who observed a tendency for higher numbers of bovine oocytes aspirated during the colder months and lower numbers during the hotter months. In addition, a negative correlation of this variable was found with an increasing THI. These results are possibly related to an affectation in the follicular dynamics [3,42], since HS is one of the environmental factors that have more detrimental effects on ovarian function, reducing oocyte development [32].

On the other hand, ultrasound studies have provided evidence that HS alters the dynamics of follicular growth [43,44]. The present study results confirm what was reported by Gendelman et al. [45], who performed follicular aspiration in Holstein cows during the summer and found that the ovarian reserve of oocytes is sensitive to high environmental temperatures. Similarly, Wolfenson and Roth [43] found that HS alters gonadotropin secretion, which compromises the most essential functions of the ovary, such as the regulation of follicular growth and oocyte maturation and development [46]. Furthermore, HS has been reported to depress GnRH and LH secretion, compromising their functions and causing low ovulation [47]. Unlike LH, FSH secretion increases under HS conditions and is associated with more follicles growing in the ovary [11,48]. Thus, the variability in hormonal secretion can be a crucial point in explaining the alterations in oocyte development in the different months of the year.

### 4.2. Oocyte Viability

Both cows and heifers were subjected to HS during most months of the year. However, the number of viable oocytes did not differ among many winter, summer, and autumn months. Furthermore, the correlation between the THI and viable oocytes for both cows and heifers was moderate to low. These results are puzzling because it is well documented that hyperthermia during in vitro maturation impairs various organizational processes in the oocyte, such as migration of both cortical granules and mitochondria [49]. Therefore, it has been suggested that the partial congregation of mitochondrial distribution in the heat-stressed oocytes can be due to poor intercession of mitochondrial translocation by microtubules [50,51]. Roth [42] also observed that when oocytes in vitro are exposed to temperatures greater than 41 °C, they suffer damage to the cytoplasm. Another possible cause of the results of this variable is because the viability of the oocytes is subjective and depends on the experience and criteria of the evaluator. Although in the present study it was the same expert technician who evaluated all the oocytes, which guarantees the reliability of the results, only the visual morphology criterion was used for their selection. Wang and Sun [51] indicate that the visual morphology technique is not so exact, which could give a variability of results in the evaluation of this variable; therefore, we suggest that more reliable techniques should be developed with more precise selection criteria of viable oocytes to achieve greater accuracy for maximizing IVEP.

### 4.3. Oocyte Developmental Competence

In cows, a lower oocyte developmental competence was observed from June to September, and in heifers, this was observed from June to August. This result is probably because heifers are comparatively heat resistant due to less production of metabolic heat and higher heat dissipation efficiency, despite the fact that heifers were not cooled throughout their growth phase. In the case of lactating multiparous cows, the body temperature increases with a high ambient temperature, and they could not dissipate body heat adequately to maintain thermal equilibrium [52]. This heat accumulation would result in compromised reproduction function [43].

In heifers, oocyte developmental competence was negatively affected by an increasing THI, although this association was weak. In the case of the cows, the association between these variables was stronger. In heifers, oocyte developmental competence did not necessarily decrease in most months, which may indicate that heifers are more tolerant of HS than mature cattle, although they still suffer from HS to some degree. To acclimatize to HS, heifers experience a series of physiological and metabolic changes to achieve the redistribution of energy, from which the compromised growth performance follows [53]. This may be related to the metabolic expenditure to which cows are exposed due to the high production of the ongoing lactation [54]. In relation to this, Roth [46] found that lactating cows have less capacity to dissipate heat. On the other hand, Sartori et al. [55] documented that cows with higher milk production have a low efficiency in embryonic development, and consequently, a lower oocyte developmental competence than non-lactating cows. The present study’s results agree with Gendelman et al. [45], who observed a decrease in the oocyte developmental competence of oocytes collected from Holstein cows in the summer and a delayed division in the first embryonic divisions. Likewise, Roth [46] found a low proportion in the development of embryos until the blastocyst stage in heat-stressed cows. On the other hand, it has been shown that the mechanisms by which severe HS affects oocyte competence involve cellular and molecular damage [56]. This leads to a failure in the maturation and fertilization of the oocytes. Furthermore, high temperatures (41 °C) during the germinal vesicle stage reduce the meiosis and metaphase II stage in embryos [12].

### 4.4. Embryo Production

The embryo production obtained in the present study is comparable to the 2.1 embryos per OPU-IVEP procedure for Holstein cows [57]. Still, it is much higher than values reported by Rust et al. [41], who never reached >0.5 embryos per collection in a commercial embryo production program using OPU. There was a marked reduction in embryo production in the warmest months; these differences were more significant in cows than heifers. According to Roth [58,59], two to three estrous cycles are needed to recover the quality of oocytes damaged by high ambient temperatures. It is worth noting that hyperthermia of oocytes also hampers embryonic development, even in the absence of subsequent HS [3], which compromises fertility [60].

In heifers, the effect of HS in summer on embryo production was not as clear as in cows. A possible explanation is the greater HS tolerance capacity and greater adjustments in their thermoregulation during HS than lactating cows [55]. The lower embryo production derived from oocytes harvested in the summer months is apparently due to the direct effects of HS on the embryo [54], which triggers mechanisms that reduce its survival. Roth [40] and Sakatani [60] found a lower production of in vitro embryos in the warmer months compared to the cold months.

Embryos in the early stages of development are more sensitive to HS [19] than during the blastocyst stage [59]. This is possibly due to the activation of the embryonic genome that occurs in the early stage of embryonic development and after it allows the embryo to develop thermotolerance [51]. Another possible cause of low IVEP in months with high temperatures is due to a low expression of the growth differentiation factor gene (GDF9; essential for the early development of the embryo) [48], since according to Ferreira et al. [61], during the summer there is a lower expression of this gene in the maturation of in vitro oocytes obtained from both heifers and lactating cows.

## 5. Conclusions

These results reaffirm that high-yielding dairy cows in a hot-arid environment at 25 °N are susceptible to heat load, which is reflected in the lower number and quality of oocytes harvested by ovum pick up, and the in vitro resultant embryos. The effect of month and the temperature–humidity index on oocyte recovery, quality, and in vitro embryo production is an important finding that affects the efficiency of this technology in the cattle industry. This information can be used to plan future in vitro embryo production programs for dairy cows. This strategic planning should enhance the results obtained with this technique and make the use of this procedure more cost-effective.

## Figures and Tables

**Table 1:** Temperature and THI averages for the months in which the experiment was carried out.

Months	Average (°C)	Maximun	Minimun	Average (THI)	Average Relative Humidity
January	14.65 (±2.13)	29	1	74	34
February	14.85 (±2.77)	28	2	73	35
March	20.79 (±1.94)	35	7	79	27
April	21.80 (±1.55)	30	5	76	19
May	27.42 (±1.56	42	10	90	24
June	29.30 (±1.34)	39	17	88	31
July	28.88 (±1.24)	39	20	86	40
August	27.94 (±1.27)	38	16	90	42
September	24.81 (±1.48)	37	15	84	42
Octuber	22.03 (±1.55)	33	11	83	49
November	17.83 (±1.86)	34	2	80	57
December	11.93 (±1.58)	27	−5	79	56

## Data Availability

Data are contained within the article.
